# Heat waves elevate risks of airway hypersensitivity that inhaled endogenous ions reduce

**DOI:** 10.21203/rs.3.rs-6839331/v1

**Published:** 2025-10-06

**Authors:** David Edwards, Brian Button, Aurelie Edwards, Kian Fan Chung, Hong Dang, Mark Gutay, Nick Griffin, Justin Gerenza, Linying Wang, Hisham Abubakar-Waziri, Deen Bhatta, Dennis Ausiello, Dan Li

**Affiliations:** JOHNS HOPKINS UNIVERSITY MEDICAL SCHOOL; University of North Carolina at Chapel Hill; Boston University; National Heart and Lung Institute, Imperial College London; University of North Carolina at Chapel Hill; University North Carolina; University North Carolina; University North Carolina; Chinese Academy of Sciences; Imperial College London; Sensory Cloud Inc; MGH, Harvard Medical School; Boston University

## Abstract

Heat wave burden is rapidly rising relative to pre-industrial levels and exacerbating human respiratory illnesses. Yet, how to protect human airways, short of deep and sustained reductions in greenhouse gas emissions, remains unclear. Here, we find by climate modeling, continuum mechanics analysis, in vitro cell culture models, and human clinical data, that heat waves increase risks of cough hypersensitivity, which can be reduced by alkaline aerosols of endogenous salt ions. We predict that the mouth breathing of indoor and outdoor air during heat waves, by exposing human airways to aridity, causes mucosal collapse onto cilia and inflammatory compression of airway epithelial cells. Using airway lining interface cultures of human bronchial epithelial cells, we find that mucosal collapse alters the expression of genes associated with airway hypersensitivity, including TRPV4, PIEZO1, SCNN1A, SCNN1B, ANO1, and CFTR. Our theoretical and experimental models indicate that mucosal collapse can be reversed by inhalation of alkaline (pH > 8) hypertonic divalent salts for 4–6 hours by globular protein translocation toward the airway epithelium. We find, on analysis of cough recordings in a recently published clinical trial, that inhaling alkaline hypertonic divalent salt aerosols with pH > 8 every 4–6 hours significantly reduced placebo-adjusted cough bouts (34%) (n = 8) (p = 0.01) among refractory chronic cough patients. Inhaled aerosols of the same composition with pH < 8 had no effect on cough bouts (−12%) (n = 4). Aerosols with endogenous ions may help reduce global warming threats to human respiratory health.

## Introduction

Frequency of extreme weather events is rising with global warming^1^ and worsening symptoms of respiratory illness, including asthma,^2^ chronic obstructive pulmonary disease (COPD),^3^ influenza,^4^ and chronic cough.^5^ Impacting urban more than rural settings,^6^ extreme heat and cold waves^7^ are hastening the atmospheric risks of global warming for inner city children, who are among asthmatics most at risk of asthma morbidity and mortality.^8^

Hot and cold air, similar to air at altitudes too high for survival of most plant matter,^9^ expose human airways to high vapor pressure deficit (VPD),^10^ a measure of atmospheric aridity that is defined by the difference between water saturation pressure and vapor pressure at a given temperature, pressure and relative humidity condition.^11^ At VPD levels (1.5 kPa) above which most plant leaves wilt,^12^ airway mucus rapidly thins and collapses onto cilia.^10^ Above VPD levels (3.0 kPa) at which the most heat-resistant plants die,^13^ airway mucosa compress airway epithelial cells with pressures exceeding 1 kPa, promoting the acute secretion of inflammatory cytokines, including IL6, IL33, and TNFα,^10^ and triggering an inflammatory cascade implicated in the pathogenesis of chronic respiratory disease.^14^

Mechano-sensitive ion channels (MSC) play a prominent role in this airway inflammatory response.^15^ MSCs such as TRPV4,^16^ PIEZO1,^17^ and ENaC^18^ respond to compression by elevating the secretion of ATP^19^ and IL6^20^ in the case of TRPV4, chemokine secretion such as CXCL2^21^ in the case of PIEZO1, and sodium ion permeability^22^ in the case of ENaC.^23^ Up- and down-regulation of these and other MSCs, including CFTR and AQP5, is common to conditions of airway hypersensitivity,^24^ and accompanies heightened tendency for cough^25^ and bronchospasm.^26^

Airway epithelial cell compression in extreme atmospheres occurs by elevation of osmotic pressure in airway surface liquid (ASL), a principal contributor to which is globular protein.^27^ Hundreds of globular proteins diffuse within ASL,^28^ moving from mucus into the periciliary layer (PCL) given their small size (20 kDa to 200 kDa) relative to mesh size between tethered mucins (MUC1, MUC4, MUC16). Globular proteins also associate with the primary gel-forming mucin macromolecules MUC5AC and MUC5B, in what has been labeled the “mucin interactome.”^29^ Held together largely by electrostatic and weak hydrophobic interactions, the mucin interactome contains ~ 30% of total globular protein in healthy hydrated human ASL.^29^ This sequestration changes with evolution of mucin concentration and ionic environment, altering mucosal viscoelastic, antimicrobial, and antioxidant immune function.^29^ Evolution of globular protein sequestration following mucin concentration changes appears to occur gradually, over time scales ~ 30 minutes based on measured mucin/globular protein absorption and desorption times,^29^ while relatively instantaneous with alterations in local ionic environment, such as abrupt changes in pH.

Dehydrated airways can be rehydrated by the inhalation of hyperosmolar aerosols,^30^ as is commonly practiced in the treatment of cystic fibrosis (CF).^31^ Hypertonic aerosols such as hypertonic saline (HS) and mannitol^32^ increase rates of mucociliary clearance (MCC)^33^ by lifting mucus off compressed cilia for durations that can vary from ~ 30 mins to several hours as a function of mucin, globular protein concentration, and sodium, chloride and water membrane permeabilities.^34^ In hypersensitive airways, HS and mannitol can also trigger cough and bronchoconstriction.^35,36^ Hyperosmolar aerosols with cell-permeating cation or lacking cell-permeating anion, such as NaCl and mannitol, provoke acute peaks of intra-cellular sodium and depressions of extracellular chloride,^37^ inducing acute acidification,^38,39^ and possibly triggering cough by TRPV1 activation.^40^ Hypertonic divalent salts (HDS), such as endogenous MgCl_2_ and CaCl_2_, avoid acidification provocation for these same reasons,^37^ hydrate for longer periods of time owing to the slow paracellular egress^37^ of the divalent cations Mg^++^ and Ca,^++^ and actually appear to treat cough in hypersensitive airways.^41^

We wished to explore the mechanism of action and potential therapeutic value of HDS aerosols for rehydrating airways otherwise dehydrated on exposure to extreme atmospheres. We hypothesized that HDS aerosols with pH above the pKa of the cysteine disulfide linkages of MUC5AC and MUC5B macromolecules (8.3 in solution, while ranging from 7.4 to 9.1 in physiological environments)^42^ would partially and reversibly dissolve the mucus hydrogel.^43^ We assumed this dissolution would dissociate the mucin interactome, liberate globular proteins to play their natural role as osmotic pumps, and thereby provide greater and prolonged hydration relative to HDS aerosols with relatively low pH. We further hypothesized that, by prolonging airway hydration, alkaline HDS rehydration would reduce compression for prolonged periods of time, and thereby enable a potentially practical and safe therapy for treating diseases of airway hypersensitivity provoked by the breathing of dry air.

We used climate model simulations to estimate the rising risk of human airway exposure to high (> 1.5 kPa) and ultra-high (> 3.0 kPa) VPD during extreme heat events in the United States from 1980 to 2019. We used continuum mechanics to analyze the response of airway mucosa to such high VPD levels before and after topical deposition of HS and HDS aerosols of variable pH. We used airway liquid interface (ALI) cultures of human bronchial epithelial (HBE) cells to study gene expression associated with cough hypersensitivity on exposure to air with high VPD, and the rehydration behavior of the ALI cultures in response to topical deposition of HS and HDS aerosols of variable pH. We finally analyzed cough bout incidence in a recent exploratory clinical study of 12 refractory chronic cough patients following daily inhalation of HDS aerosols with pH above and below pH 8. The results of our studies are reported here.

## Results

### Exposure of human airways to inflammatory VPD is rapidly rising due to heat waves

**Figure 1:**
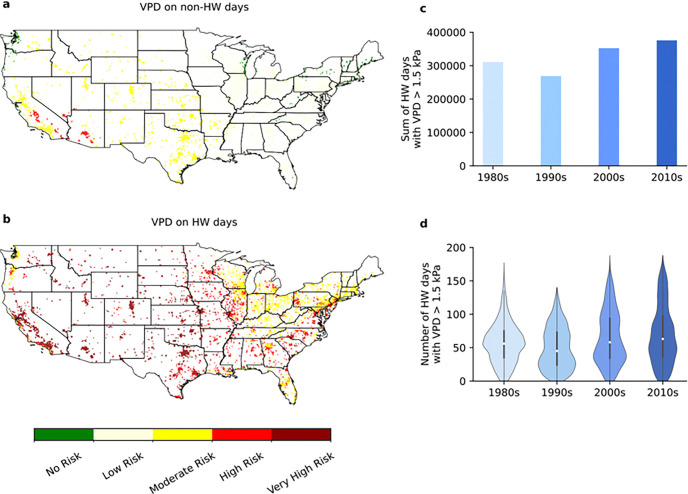
Extreme heat event and VPD exposure in the continental United States from 1980 to 2019. Projections of environmental (outdoor air) VPD over urban land for the continental US over the period 1980–2019 based on a climate model simulation carried out using CESM version 2.1.3 as further described in the Methods. A city must be larger in terms of surface area fraction than 0.1% of the grid cell to be included. The grid cell size is about 12.5 km (1/8 degree). In total, there are 5410 cities considered here. Average VPD in summer months (June, July, August) on (a) non-heat-wave (non-HW) days and (b) heat-wave (HW) days. Dark green denotes no risk (VPD < 750 Pa), light yellow denotes low risk (750 Pa < VPD < 1.5 kPa), dark yellow denotes moderate risk (1.5 kPa < VPD < 3.0 kPa), red denotes high risk (3.0 kPa < VPD < 4.5 kPa) and dark red denotes very high risk (VPD > 4.5 kPa). The VPD values reported here are for the urban part of the grid cell (i.e., for the urban land). (c) The total number of HW days with VPD > 1.5 kPa per decade summed over the simulated 5410 cities over the continental US. (d) The distribution of HW days with VPD > 1.5 kPa per decade across the simulated 5410 cities over the continental US. The shape of each violin represents the distribution of HW days, with the internal box indicating the interquartile range and the central white dot indicating the median value.

We assessed the risk of human airway exposure to inflammatory dry air during extreme heat events over cities in the United States by performing a land-only simulation in the historical period (1980–2019) for the continental United States (see Methods). Our findings are shown in Figure 1.

During summer season (June, July, August), VPD on non heat-wave (non-HW) days was primarily of *low inflammatory risk* (66.4%, Fig. 1A), typical of the entire United States over this same period of time.^10^ In comparison, VPD on heat-wave (HW) days was primarily of *high-to-very high inflammatory risk* (60.0%, Fig. 1B), typical of the entire United States at the end of the 21st century with current global warming estimates corresponding to RCP8.5.^10^ The total number of HW days with VPD > 1.5 kPa, summed over the simulated 5,410 cities (Fig. 1C), increased from 310,805 in the 1980s and 268,888 in the 1990s to 352,305 in the 2000s and 375,816 in the 2010s. HW days over all cities were unevenly distributed, as illustrated by the shapes of the violin plots in Fig 1D. The number of cities experiencing the median number of HW Days in the 1980s actually diminished in the 1990s, 2000s, and 2010s (Fig 1D). This occurred as a consequence of an increasing number of cities each decade with over 100 HW Days. Cities with greater than 125 HW Days in the 1980s and 90s (125) multiplied 10 fold by the 2000s and 2010s (1271) (Fig 1D). In terms of the severity of heat stress experienced by the US population on these HW Days, average VDP in the ten most populous US cities (New York City, Los Angeles, Chicago, Houston, Phoenix, Philadelphia, San Antonio, San Diego, Dallas, Jacksonville) remained consistently ~ 3 kPa across the four decades 1980–2020. Over these same decades average VPD in the ten hottest cities per decade reached ~ 6 kPa.

### Rehydration of dehydrated airway mucosa occurs in pH-dependent manner

**Figure 2:**
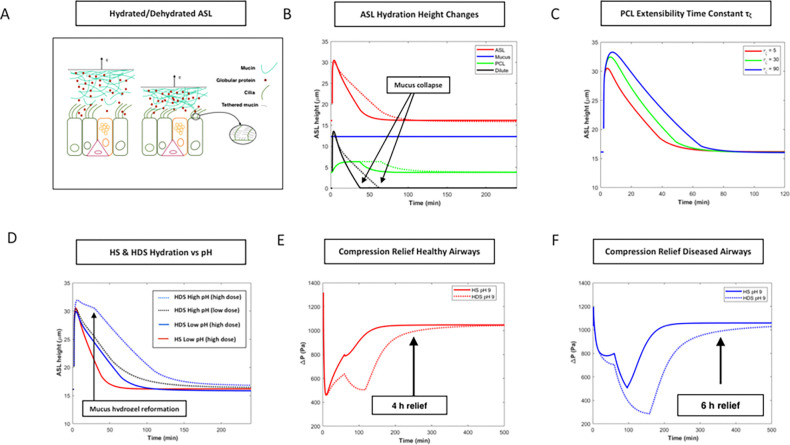
Rehydration of human airways post exposure to aridity. Rehydration of dehydrated airway surface liquid (ASL) by hypertonic salt aerosols with variable pH alters ASL structure and alleviates the compression of airway epithelial cells caused by exposure to dry air atmospheres. (A) Cross section of a healthy ASL above a human airway epithelium comprising ciliated epithelial cells, goblet cells and basal progenitor cells. Mucin tethered to cilia and the apical epithelial membrane and globular protein provide the principal osmotic pressure of the ASL in steady state. On exposure to warm humid air, characteristic of slow nasal breathing, the ASL is fully hydrated. On exposure to arid conditions, the mucus layer thins and collapses on cilia. (B) Change in the heights of ASL, mucus hydrogel, dilute mucin layer, and PCL vs time post deposition of HS (NaCl, 4.3% w/w, pH 7) (solid lines) and HDS (MgCl_2_, 4.3% w/w, pH 7) (dashed lines) with total deposited mass 25% of ASL mass. Airway generations 0 to 6 in normal tidal breathing (15 L/min), or generations 0 to 9 with fast breathing (30 L/min), are assumed dehydrated by the mouth breathing of 25°C, 30% RH air, resulting in exposure to an average VPD ~ 1.5 kPa (ranging from ~ 3.4 kPa in the larynx to ~ 0.5 kPa in the first bronchioles). (C) Change in the height of ASL following deposition of HS (NaCl, 4.3% w/w, pH 7) versus cilia extensibility time $\tau_{\zeta^{2}}$. (D) ASL height post deposition of HS and HDS aerosols at low $\left(K_{PCL-0}=0.7\right)$ and high pH $\left(K_{PCL-0}=3.0\right)$. (E) Epithelial cell compression post alkaline HDS (MgCl_2_, 4.3% w/w, pH 9) deposition versus HS (NaCl, 4.3% w/w, pH 9) for “healthy airways” with fast cilia extensibility (*τ*_*ζ*_ = 5 mins) and normal mucus thickness (*h*_0_ = 20 μm). (F) Epithelial cell compression post alkaline HDS (MgCl_2_, 4.3% w/w, pH 9) deposition versus HS (NaCl, 4.3% w/w, pH 9) for “diseased airways” with slow cilia extensibility (*τ*_*ζ*_ = 30 mins) and hyper-secreted mucus thickness (*h*_0_ = 40 μm). See Methods.

We used continuum mechanics (Methods) to analyze airway mucosal collapse and reversal as a consequence of heat waves. As base case, we assumed human airway exposure to the minimal heatwave condition (VPD ~ 1.5 kPa), typical of the mouth breathing of cooled indoor air or warm outdoor air on a HW day (Supplemental Note 1) (Fig 2A). Chronic mouth breathing, recently estimated to afflict 40–50% of children^45^ and adolescents,^46^ induces a base case average evaporation rate q sufficient to thin airway mucus from the larynx to bronchiole generation 6 (Supplemental Note 1).^10^ Mucosal thinning accompanies collapse onto cilia within the trachea, primary, secondary and tertiary bronchi in this same base case. Mucosal collapse compresses the dehydrated airway epithelial cells by an amount (Methods)

(1) $$\Delta P_{d}=\frac{q}{L_{p}}-K_{PCL-d}\Delta \Pi_{gp-0}-\Delta \Pi_{tm-0}$$

with $L_{p}$ is the hydraulic permeability of the apical epithelial cell membrane, $\Delta \Pi_{gp-0}$ and $\Delta \Pi_{tm-0}$ are the osmotic pressures of globular protein (~ 350 Pa) and tethered mucin (~ 180 Pa) in the fully hydrated “0” state, and $K_{PCL-d}$ the partition coefficient of globular protein from the dehydrated mucus into the PCL. In the base case scenario (atmospheric condition 25°C, 35% RH), it follows that, on average within the larynx, trachea and through the 5th airway bronchiole airway (Supplemental Note 1), VPD ~ 1.5 kPa, $K_{PCL-d}=0.44$, Δ*P* = 0.98 kPa. On exposure to the heatwave extreme characteristic of the 10 hottest cities in the United States, atmospheric VPD ~ 6 kPa with average airway VPD ~ 2.7 kPa in these same airways, $K_{PCL-d}=0.19$ and Δ*P* = 2.68 kPa. Thus, tidal mouth breathing during heat waves compresses airway epithelial cells above the acute kPa threshold that has been implicated in the provocation of cough^25^ and bronchospasm.^26^

We examined numerically the consequence of rehydrating dehydrated human airways by topical deposition of non-alkaline HS (NaCl) and HDS (MgCl_2_) aerosols (Fig 2B). Following deposition, a dilute mucin layer immediately grows above the PCL (black lines), lifting dehydrated mucus off compressed cilia, allowing the PCL (green lines) to expand on an extensibility time scale $\tau_{\zeta }$ assumed ~ 5 minutes. Water uptake is negligible in the mucus layer given the relatively rapid diffusion of the least mobile constituents of the ASL $\left(I^{2}/D∼10^{-12}{\;m}^{2}/10^{-12}{\;m}^{2}/s∼1\;s\right)$ in comparison to mucus swelling time (minutes).^48^ Continued evaporation therefore retains the transpiring hydrogel mucin stratification and mucus thickness (blue line). Slow clearance of the divalent cation relative to the monovalent cation prolongs hydration for HDS (dashed lines).

**Figure 3:**
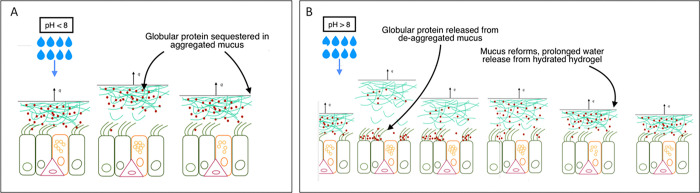
The role of the mucin interactome in the prolongation of rehydration at high pH. Inhalation of HDS aerosols with pH below above the cysteine pKa (~ 8.3) alters the osmotic availability of globular protein. (A) At relatively low pH (< ~ 8.3), deposition of the hypertonic salt aerosol lifts the dehydrated mucus hydrogel off cilia, while does not swell the mucus hydrogel. Globular protein remains highly sequestered in the dehydrated mucin interactome. Following water reabsorption and mucus collapse back onto cilia, cilia compress with no further water release from the mucus hydrogel. (B) Deposition of the relatively high pH (> ~ 8.3) hypertonic salt aerosol locally and temporarily dissolves the mucus hydrogel, releasing globular protein from the mucin interactome. The neutral to acidic pH of the ASL rapidly lowers the high pH of the deposited water mass, such that the hydrogel is restored over a time frame *T*_*M*_ that is a function of ionic, mucin and other compositional parameters. With “low” deposited masses (less than 10% ASL mass per airway generation) elevation of the ASL pH is minor, while with relatively large deposited mass (e.g. the 25% ASL mass of the base case) ASL pH rises sufficiently to increase globular protein attraction to the low pH nano-environment of the airway glycocalyx, increasing the partition coefficient ($K_{PCL-0}>0.7$). This creates greater hydration relative to the low pH case, and, once the mucus hydrogel reforms, dehydration is slowed down by the slow release of water from the fully hydrated and reformed mucus hydrogel.

The speed of dehydration post topical deposition of HS or HDS aerosols depends on the extensibility time $\tau_{\zeta }$ (Fig 2C), or severity of airway dehydration. This prolongation of hydration in severely dehydrated or diseased airways ($\tau_{\zeta }\gg 5$ minutes) owes to the greater osmotic pressure of the compressed PCL, resulting in greater water efflux into the ASL. Duration of hydration is further extended, at all extensibility time scales, with deposition of high pH HDS (Fig 3). For large deposited masses (25% ASL mass), ASL pH increase is significant, rising from ~ 7 to ~ 7.5 in the case of ASL of neutral pH. This rise in ASL pH, given the relatively acidic nano-environment of the glycocalyx^47^, is assumed to increase partitioning ($K_{PCL-0}$) of globular protein into the PCL, increasing osmotic water flow into the ASL (Fig 2D). For deposited masses less than 10% ASL mass, ASL pH rise is negligible, and the consequence of globular protein liberation from the disordered mucin interactome is a relatively modest increase in the globular protein osmotic pressure (Fig 2D). Airway epithelial cell compression falls post deposition (Fig 2E, 2F) owing to globular protein release from the disrupted mucus hydrogel. Compression relief at pH 9 is significantly longer for HDS relative to HS aerosols both for healthy (Fig 2F) and diseased airways (Fig 2G). The initial rise in compression and subsequent fall at ~ 30 minutes reflects the transition from a globular-protein-dominated to tethered-mucin-dominated compression relief around the time of reformation of the disrupted mucus hydrogel. The second rise in compression at ~ 120 to ~ 180 minutes reflects mucus collapse and subsequent slow recompression of the PCL. Overall duration of relief from the mechanical compression of epithelial cells caused by exposure to extreme indoor and outdoor atmospheres is approximately 4–6 hours depending on severity of airway dehydration (Figs 2E, 2F).

The spike in compression on deposition in Figs 2E & 2F reflects the assumed stepwise change in $K_{PCL-0}$ from 0.7 to 3. A gradual increase of $K_{PCL-0}$ over the first minutes of hydration, the more likely consequence of the natural process of mucin interactome disentanglement, combined with the rapid ASL volume growth and dilution of globular protein concentration, smooths out the compression response, favoring a more gradual diminution of compression over the first minutes of rehydration, as globular protein accumulates in the PCL post breakup of the mucin interactome (Fig 3).

### Mucosal collapse increases hypersensitivity and is reversed in pH-dependent manner

We sought to explore the impact of extreme atmospheres on human airway hypersensitivity. We therefore exposed ALI cultures of HBE cells to an extreme atmosphere of 4.4 kPa (characteristic of the average tracheal and main bronchi exposure in the 10 US cities with worst heatwave incidence in recent years) for 8 hours in a humidity-controlled confocal microscope chamber with air circulation (Methods). As control, we exposed ALI cultures of HBE cells to a highly humidified atmosphere of 95% RH (VPD = 0.5 kPa) After 3 hours of exposure at the 30% RH condition, or at both the 30% and 95% RH conditions after 8 hours of exposure, cells were withdrawn, RNA extracted, and bulk RNA-sequence data analyzed computationally (Methods). We assessed expression of the following genes potentially associated with airway hypersensitivity — ANO1, AQP1, AQP5, CFTR, F2R1, ORAl1, P2RX3, P2RX4, P2RX7, P2RY2, PIEZO1, PIEZO2, SCNN1A, SCNN1B, SCNN1D, SCNN1G, TRPA1, TRPV1, TRPV2, TRPV3, TRPV4, TRPV5, TRPV6 (Supplemental Note 3).

**Figure 4:**
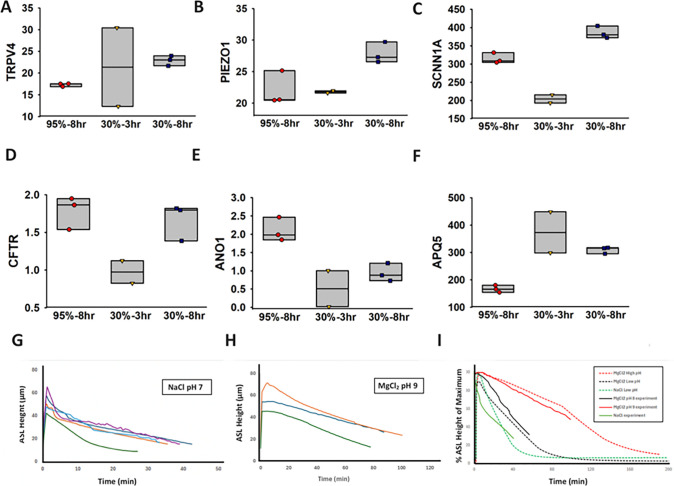
Dehydration and rehydration of ALI cultures of HBE cells. (A-C) Bulk RNA-seq gene expression results for (A) TRPV4, (B) PIEZO1, (C) SCNN1A, (D) CFTR, (E) ANO1, F (AQP5) following exposure of ALI cultures of HBE cells to air at 37°C and 95% RH and 30% RH (n=3) for 8 hours. (G, H). ASL height versus time following nebulization of (G) HS (4.3% w/w NaCl) (n=5) and (H) HDS (4.3% w/w MgCl_2_) pH 9 (n=3) measured at 6 lateral points in the plane of the confocal microscope lens. (I) ASL height normalized by peak ASL height following nebulization of HS (4.3% w/w NaCl) and HDS (4.3% w/w MgCl_2_) pH 8 and pH 9. Theoretical predictions are based on the model parameters of Fig 2 with *τ*_*ζ*_ = 5 minutes and mucus reformation time (*T*_*M*_) = 90 minutes.

Figures 4A–F present results for 6 MSC genes highly expressed in airway epithelial cells. TRPV4 (p=0.009) and PIEZO1 (p=0.0432) gene expression rises significantly at 8h dry-air exposure compared to the reference 95% RH condition (Figs 4A, 4B), as is consistent with reported TRPV4 and PIEZO1 up-regulation following mechanical compression of epithelial cells.^48, 49^ No significant change is either gene is observed at 3 h of exposure (p>0.05). SCNN1A (Fig 4C) falls after 3h at 30% RH (p=0.0105) relative to 95% RH, while after 8h arid exposure SCNN1A expression rises (p=0.0057) relative to the humid condition. These same trends are observed for both α and β subunits of ENaC (i.e., SCNN1A, SCNN1B, see Supplemental Note 3). Down-regulation of ENaC appears to be a natural defense against dehydration, and particularly against down-regulated CFTR,^50^ as occurs (Fig 4D) at 3h of exposure (p=0.0439). ENaC expression rises from 3h to 8h consistent with reports of up-regulation of ENaC under mechanical stress,^51^ suggesting that the immune defense of the airways to dehydration is surpassed with prolonged dehydration > 3h. The relatively small rise in CFTR expression at 8 hours (CFTR is unchanged at 8 relative to 95% RH, p = 0.592), as well as the fall in CFTR observed at 3 h in the 30% RH condition (Fig 4D), appears to reflect CFTR down-regulation on the prolonged ATP exposure^52^ consequent to TRPV4 compression.^48^ TRPV4-mediated ATP secretion further triggers cough by stimulating P2X3 receptors, which also up-regulate at 8 h of dry air exposure while not at 3 h of exposure (Supplemental Note 3).^53^ Expression behavior of the ANO1 chloride channel gene (Fig 4E) parallels the behavior of CFTR, whereas the up-regulation at 8 h of aridity exposure (p=0.0001) of AQP5 gene, encoding one of the two principal water channels in airway epithelial cells, appears to be a direct response to the dehydrated state (Fig 4F).

We explored whether rehydration of dehydrated ASL by hypertonic salts would follow the predicted behavior (Fig 2). We therefore exposed ALI cultures of HBE cells to air at 37°C and 60% RH (VPD = 2.2 kPa) reflective of the Fig 2 base case. We exposed the ALI cultures to this atmosphere for 1 hour in a humidity-controlled confocal microscope chamber with air circulation. We then nebulized onto the dehydrated ASL HS (4.3% w/w NaCl) pH 7 and HDS 4.3% w/w MgCl_2_) pH 8 and 9. We monitored ASL height over time as described in the Methods and our results are shown in Fig 4G–I. Since mucus height varied from sample to sample (Figs 4G and 4H), we normalized each set of data by the maximum ASL height achieved and compared the normalized data as shown in Fig 4I. Also shown are the theoretical predictions based on a PCL extensibility time scale $\tau_{\zeta }=5$ minutes, consistent with the acute nature of the dehydration of healthy epithelial cells.

### Rehydration of hypersensitive airways reduces cough bouts in pH-dependent manner

We hypothesized that prolonged relief of airway epithelial cell compression by alkaline HDS aerosols would result in greater reduction of cough hypersensitivity in refractory chronic cough (RCC) patients by aerosols of HDS pH > 8 relative to HDS pH < 8 on inhalation every 4 to 6 hours, or 4 times per day. We therefore evaluated cough bout frequency in a recent exploratory clinical trial conducted by our group^54^ with RCC patients treated by nasal inhalation of alkaline HDS pH > 8 (n=8) or non-alkaline HDS pH < 8 (n=4) aerosols and a nasal saline control. Previously, we reported daily cough rate response.^54^ Here, we report on cough bout response, with cough bouts defined^55^ as 2 or more consecutive coughs with 2 s or less interval and monitored continuously using a Hyfe digital cough watch monitor.^56^ Patients self-administered the aerosol 4 times per day by nasal inhalation and used an identical inhaler for 7 days of active and 7 days control treatment. Cough bout rate was monitored continually for a week of baseline cough assessment, a week of control administration, and a week of active treatment.

**Figure 5:**
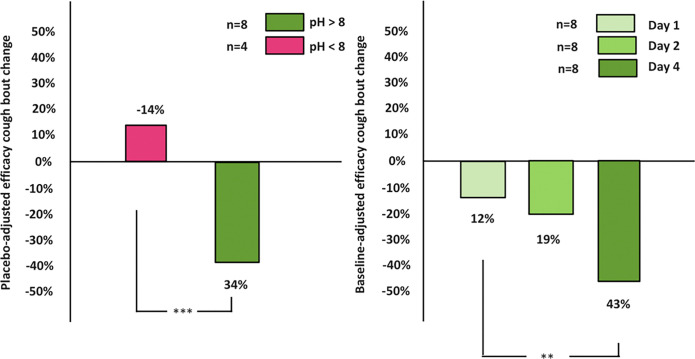
Suppression of cough bouts by the daily inhalation of HDS aerosols with pH 8 and pH 9. Twelve refractory chronic cough patients participated in an exploratory, single-blinded, nasal-saline-controlled study to examine cough-suppression efficacy of an alkaline HDS composition (4.9% w/w) at pH < 8 or pH > 8 self-administered by nasal inhalation 4 times a day using a hand-held soft-mist inhaler. Each subject was monitored continuously using a digital cough monitor watch for one week of baseline, one week of control treatment, and one week of active treatment. Baseline daily cough rates ranged from 4 to 34 coughs/hour with mean visual analog score 65±17 pre- and post-baseline testing.(A) Placebo-adjusted efficacy of cough bout suppression from Day 2 of active treatment for HDS pH 8 (n=4) and HDS pH 9 (n=8). *** = p < 0.001. (B) Daily cough bouts post deposition of HDS with pH > 8 relative to baseline cough bouts for Days 1, 2 and 4 (n=8). ** = p < 0.01.

Relative to placebo, cough bout frequency diminished by 34% (p=0.01) for those subjects who self administered the alkaline HDS pH > 8 aerosols from Day 1 of treatment (Fig 4A), while no suppression of cough bout frequency occurred (− 14%) (p=0.22) for those who self administered the pH < 8 aerosols (Fig 5A). Suppression in cough bout frequency relative to baseline increased (p=0.01) from 12% on Day 1 to 42% on Day 4 for the alkaline HDS pH 9 treated patients with no change in cough bout frequency over this same timeframe for the alkaline HDS pH 8 patients (Fig 5B).

## Discussion

Heat waves elevate incidence of cough and bronchospasm, increase hospitalization rates of asthmatics and sufferers of COPD,^57^ and threaten the respiratory health of vulnerable populations, including children,^58^ the elderly,^59^ and low-income populations.^60^ The unprecedented impact of heat burden on the human population is rapidly growing, and estimated to afflict between 52% and 92% of humans born today by the end of this century based on a 1.5°C to 3.5°C rise in global temperatures.^1^

Heat waves expose human airways to high levels of aridity during chronic mouth breathing.^10^ We have found that, in recent decades, heat wave exposure in urban centers of the United States ranged from from VPD ~ 3 kPa in the ten largest cities to VPD ~ 6 kPa in the ten hottest cities (Fig. 1). Exposing human bronchial epithelial cells to an atmosphere in this range (4.4 kPa) causes mucosal collapse, compression of airway epithelial cells, and up-regulates TRPV4 (Fig. 3A), PIEZO1 (Fig. 3B) and SCNN1A (Fig. 3C) following 8 hours of exposure, while not following 3 hours of exposure. Up-regulation of these genes increases the likelihood that relatively small mechanical provocations result in relatively large secretions of ATP (TRPV4), inflammatory cytokines (TRPV4, PIEZO1) and airway acidity (SCNN1A), thereby increasing the likelihood of airway hypersensitivity on the breathing of hot atmospheres.^58,59^ While less severe, inflammatory compression of airway epithelial cells also appears to occur in common indoor atmospheres, and particularly on the mouth breathing of typical indoor, air-conditioned air in the summer, or heated indoor air in the winter (VPD ~ 1.5 kPa) (base case of Fig. 2).

Relief from the inflammatory compression accompanying mucosal collapse appears to occur for less than 3 hours on the inhalation of non-alkaline HS and HDS aerosols (pH < 8), while greater than 3 hours following the inhalation of alkaline HDS aerosols with pH > 8 (Figs. 4I, 3D–F). We attribute this prolonged hydration to the reversible breakup of the mucin interactome at high pH, combined with slow clearance of the divalent cation (Mg^++^ or Ca^++^) (Fig. 3, Supplemental Note 4). Similarly prolonged hydration has been observed in ALI cultures of HBE cells following topical deposition of hypertonic non-alkaline HS aerosols via blockage of ENaC or on stimulation of AQP5.^34^ Within the pH excursion post deposition of the HDS pH 9 aerosol ( ~ + 0.5), ENaC permeability appears to diminish by ~ 15%,^61^ insufficient to account for the prolonged hydration observed (Fig. 3I) (Supplemental Note 4), while AQP5 permeability is unchanged.^62^ Relatively long hydration times have also been observed to occur in highly concentrated CF-like ALI cultures,^34^ and attributed to the elevated (globular-protein-dominated) osmotic pressure inherent to highly concentrated ALI cultures of HBE cells.^63^

Pig gastric mucus has been reported to dissolve at pH above 8.5, near the pKa of cysteine that forms the primary amino acid linkages between MUC5AC and MUC5B macromolecules in mucus hydrogels.^64^ Our continuum mechanic analysis (Methods) predicts that, by such a phase transition in the vicinity of the deposited aerosol, globular proteins are freed from the mucin interactome to function as freely-diffusing osmotic pumps (Fig. 2D). While globular protein concentrations in healthy ASL are far lower than those observed in CF-like airway mucosa, deposition of the alkaline hypertonic aerosol appears to lead to an accumulation of globular protein near the airway epithelium, achieving local concentrations similar to the hyper-secreted mucin ASL of CF-like airways. We postulate that this accumulation owes to temporary elevation of ASL pH relative to the acidic environment maintained in the vicinity of the glycocalyx (Fig. 3). Osmotic water flow into the ASL therefore increases to the degree experimentally observed (Fig. 4I, see also Supplemental Note 4), sustaining compressive stress release for approximately 4 to 6 hours depending on the degree of dehydration (Figs. 2F, G).

Our theoretical (Fig. 2) and experimental (Fig. 4) findings are consistent with the clinical finding that cough bouts are significantly suppressed in the hypersensitive airways of RCC human subjects following inhalation every 4 to 6 hours of HDS aerosols with pH > 8 (n = 8), while not for HDS aerosols with pH < 8 (n = 4) (Fig. 4A). The significant increase in cough bout suppression efficacy observed from Day 1 to Day 4 (Fig. 4B) in the recent clinical trial in RCC patients^54^ is also consistent with our observations (Fig. 3A–F) that MSC-related airway hypersensitivity is dependent on airway hydration state.

Many avenues of research remain to be explored. Mucin interactome evolution in extreme atmospheres likely impacts airway immune function in multiple ways, ranging from MSC activation to anti-microbial function. Molecular, cellular, bacterial and viral interactions and consequences to the pathogenesis of respiratory disease should all be investigated. Alkaline endogenous ion treatments for other diseases of the airways, including asthma, allergic rhinitis, and bronchiectasis should be explored, as well as non-endogenous compositions that might improve on the outcomes of endogenous therapies, possibly providing longer action and more profound relief from the compressive inflammatory state. The potential prophylactic benefit of managing airway hydration during heat waves by the daily inhalation of non-alkaline and alkaline endogenous ion aerosols should also be investigated, as well as acute relief treatments following wildfire or burn-pit exposure.

Heat wave impact on airway hypersensitivity mirrors heat-wave impact on crop failure rate.^1^ High VPD exposure promotes inflammatory compression of cellular matter in each system, with crop failures increasing by around 10% in heat waves,^67^ and adult asthmatic hospitalization rate by around 16%.^68^ Topical hydration will be necessary to avoid the 3–4 fold increase in crop failures predicted to occur with current climate forecasts over the course of this century.^1^ For similar mechanistic reasons, airway hydration management and treatment strategies may be critical to avoid a crisis in human respiratory illness.

## Supplementary Material

Supplementary Files

This is a list of supplementary files associated with this preprint. Click to download.

• SIAHHeatWave.pdf
